# Sedation, Analgesia, and Muscle Relaxation During VV-ECMO Therapy in Patients With Severe Acute Respiratory Syndrome Coronavirus Type 2 (SARS-CoV-2): A Single-Center, Retrospective, Observational Study

**DOI:** 10.3389/fmed.2021.762740

**Published:** 2021-12-17

**Authors:** Fang Wu, Mingna Li, Zhongwei Zhang, Jiawei Shang, Yong Guo, Yingchuan Li

**Affiliations:** ^1^Department of Critical Care Medicine, Shanghai Jiao Tong University Affiliated Sixth People's Hospital, Shanghai, China; ^2^Department of Critical Care Medicine, Tongji University Affiliated Shanghai Tenth People's Hospital, Shanghai, China

**Keywords:** VV-ECMO, SARS-CoV-2, sedation, analgesia, muscle relaxant

## Abstract

**Objective:** The pharmacokinetics and pharmacodynamics of ECMO-supported sedative, analgesic, and muscle relaxants have changed, but there are insufficient data to determine the optimal dosing strategies for these agents. Sedation, analgesia and muscle relaxation therapy for patients with severe acute respiratory syndrome coronavirus type 2 (SARS-CoV-2) receiving ECMO support are more specific and have not been fully reported. This study observed and evaluated the use of sedative and analgesic drugs and muscle relaxants in SARS-CoV-2 patients treated with VV-ECMO.

**Methods:** This study was a single-center, retrospective and observational study. Our study includes 8 SARS-CoV-2 patients treated with VV-ECMO in an intensive care unit at Shanghai Public Health Center from February to June 2020. We collected the demographic data from these patients and the dose and course of sedation, analgesia, and muscle relaxants administered during ECMO treatment.

**Results:** The doses of sedative, analgesic and muscle relaxant drugs used in patients with VV-ECMO were significant. Over time, the doses of drugs that were used were increased, and the course of muscle relaxant treatment was extended.

**Conclusion:** Sedation, analgesia, and muscle relaxant use require individualized titration in patients with SARS-CoV-2 who have respiratory failure and who are receiving VV-ECMO.

## Introduction

Extracorporeal membrane oxygenation (ECMO) is an important technique for the rescue of critically ill patients, and it is used as an adjunct therapy for critically ill patients with heart failure and/or severe respiratory failure. Patients with severe acute respiratory syndrome coronavirus type 2 (SARS-CoV-2) complicated with severe respiratory failure require ECMO support. It is essential that ECMO support includes a strategy for sedation, analgesia, and muscle relaxation during therapy. Sedation and excessive analgesia can lead to the delayed removal of the endotracheal tube, a prolonged time for mechanical ventilation, and the development of deep vein thrombosis. Long-term deep sedation during mechanical ventilation in patients with SARS-CoV-2 may delay the discovery and diagnosis of cerebrovascular side effects (1, 2). If the sedation and analgesia are too shallow, it will lead to patient agitation, man-machine confrontation, pipeline dropping, an imbalance of the oxygen supply and demand, an unstable ECMO flow, and other serious consequences and can even increase the risk of infection with the novel coronavirus in the doctors and nurses. It has been reported in the literature that a reasonable sedation and analgesia strategy can reduce the asynchrony between patients and ventilators and prevent man-machine confrontation (3); it can also reduce the oxygen consumption by reducing spontaneous muscle activity (4). Current ICU analgesia/sedation guidelines first advocate the assurance of adequate analgesia, minimizing sedation, preventing patient awakening, preventing delirium, and early recovery to facilitate ventilator weaning and early ICU weaning. However, these strategies are not always applicable to patients with ARDS who sometimes require deep sedation. Patients with severe ARDS are underrepresented in analgesic and sedative studies, and the currently recommended strategy may not be feasible (5).

An international study involving 394 ECMO centers showed that up to 75% of patients with VV-ECMO had deep sedation and 25% had light sedation (6). Shekar et al. reported that patients with VV-ECMO require a higher dose of sedatives relative to patients with VA-ECMO (7). Because more studies on neuromuscular blockers are conducted in ARDS patients undergoing mechanical assisted ventilation, relevant studies for ECMO-supported patients are scarce. The ELSO (The Extracorporeal Life Support Organization) guidelines recommend the use of muscular blockers when establishing ECMO circuits with intravenous intubation to avoid air embolism due to the patient's spontaneous breathing. The use of muscular blockers may be considered when the ECMO flow is unstable and is not recommended during other times (8, 9). The ELSO guidelines recommend minimal sedation, analgesia, and muscle relaxation in ARDS patients receiving ECMO support (8, 9), but whether this guideline is applicable to SARS-CoV-2 patients receiving VV-ECMO is unclear.

Due to the severe hypoxia, hemodynamic instability, multisystem involvement and strong infectious nature of SARS-CoV-2 patients, the strategies of sedation, analgesia and muscle relaxation need to be specific. This study was designed to summarize our team's experience with sedation, analgesia, and muscle relaxation in ECMO-supported patients with SARS-CoV-2.

## Materials and Methods

### Case Selection

This study reviewed 8 routine patients with SARS-CoV-2 supported by VV-ECMO who were admitted to the Intensive Care Unit of Shanghai Public Health Center from February 1, 2020 to June 1, 2020. The research protocol was approved by the Ethics Committee of the Sixth People's Hospital Affiliated to Shanghai Jiao Tong University [Approval No.: 2021-KY-094(K)].

### Patient Grouping

The 8 patients were divided into two groups according to death (*n* = 4) and survival (*n* = 4). Group A was the death group, and Group B was the survival group.

### Research Methods

The ECMO cannulation sites of the eight patients were all jugular and femoral veins. The oxygen saturation probe was routinely placed at the blood introduction end and the perfusion end to maintain a peripheral oxygen saturation SPO2 > 90% and a mixed venous oxygen saturation > 70%. All patients received VV-ECMO with a protective lung ventilation strategy of FIO2 <40%, a tidal volume of 2–4 ml/kg (ideal body weight), a platform pressure <25 cmH_2_O, and a respiratory rate of 8–10 breaths/min. The tidal volume was reduced if the platform pressure was above 25 cmH_2_O. Pressure control was often used before ECMO evacuation. If the patient's P/F ratio (PaO2/FiO2) was not good enough, priority was given to the ECMO parameters instead of the ventilator parameters.

Because of the patient's anxiety and oxygen demand, a bedside titration method was used for sedation, analgesia and muscle relaxation. Our goals for sedation, analgesia, and muscle relaxation are, first, to ensure a peripheral oxygen saturation of greater than 90%. However, we have found in practice that such patients with severe pneumonia need deep sedation and enough analgesia to maintain an adequate peripheral oxygen saturation. Therefore, we maintained the RASS (Richmond Agitation-Sedation Scale) score at less than or equal to −4 (deep sedation) and the analgesic CPOT (Critical Care Pain Observation Tool) score at <3 points during ECMO. The bispectral index (BIS) and train of four stimulation (TOF) were used to record the specific sedation and muscle relaxation values. In addition, the pupillary light reflex and pathological reflexes of the patients were observed regularly every day for a timely detection of possible cerebrovascular accidents. Once the patient's lung condition had improved and when patients were ready to be weaned from ECMO, the doses of muscle relaxants and sedative and analgesic drugs were gradually reduced until they were eventually discontinued. Then, antipsychotic drugs were added.

### Data Collection

**Figure 1:**
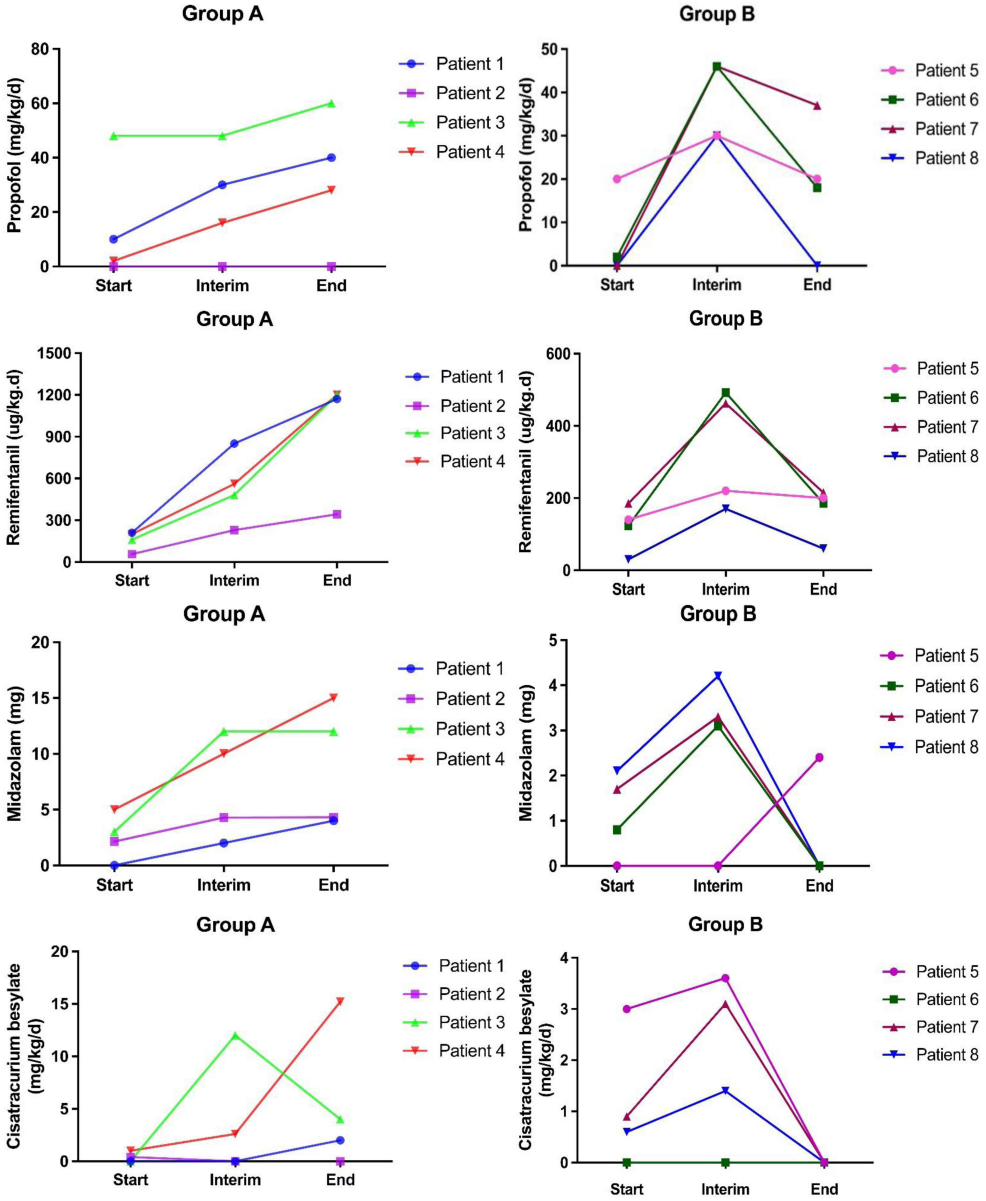
Use of sedative, analgesic and muscle relaxants during ECMO treatment: Group A was the death group, and Group B was the survival group. The usage period was divided into three main time points, namely, the ECMO start date (Start), the ECMO support interim period (Interim), and the ECMO support end date (End). The dose units used for propofol, midazolam, and cisatracurium were mg/kg/d, and those for remifentanil were ug/kg/d.

We recorded the demographic characteristics of the patients and the usage data of sedative, analgesic and muscle relaxants at three time points: the start date of ECMO (Start), the interim period of ECMO support (INTERIM) and the end date of ECMO support (END) (see Figure 1).

### Outcome

The primary study endpoint was the dose and time of sedation and the analgesia and muscle relaxation drugs given to the patient. The ECMO days, duration of mechanical ventilation (MV) and ICU stay were recorded as secondary study endpoints.

### Statistical Analyses

Consecutive variables were expressed as means ± standard deviation, and classified variables were represented by counts. All analyses were performed using SPSS 20.0. The line graph was drawn using GraphPad Prism 8.0.

## Results

### General Information

We collected 8 SARS-CoV-2 patients treated with VV-ECMO for respiratory failure: 7 males (88%) and 1 female (12%). The youngest was 25 years old, the oldest was 81 years old, and they had a median age of 64 (25, 81) years old. All eight patients had coexistent diseases, including four patients with hypertension, two with diabetes, one with coronary heart disease, one with bladder cancer, and one with chronic renal insufficiency. The risk factors in the 25-year-old patient were obesity: a body weight of 100 kg and a BMI of 33.4. The median duration of mechanical ventilation before ECMO treatment was 2 (0, 4) days in the survival group and 8 (0, 21) days in the death group. The median number of days ECMO support was 37.5 (8, 46). The P/F ratio was 96.6 (58, 155.6), and all of the patients had moderate to severe ARDS (Table 1).

**Table 1:** Patients' clinical characteristics.

**Patient no**.		**1**	**2**	**3**	**4**	**5**	**6**	**7**	**8**	**Mean ± SD**
Age (years)		64	62	65	75	25	75	63	81	63 ± 6
Gender		M	M	M	M	M	M	F	M	/
Body weight (kg)		65	65	70	80	100	75	50	70	72 ± 5
MV^a^ time (days)		4	0	4	0	0	8	21	10	6 ± 3
ECMO parameters^b^	Blood Pump Speed (rpm)	3,240	3,200	3,200	3,435	3,500	3,200	3,600	3,000	3,296 ± 70
	Blood pump flow (L/min)	4.9	3.6	3.6	4.4	4.9	4.0	4.1	3.2	4.1 ± 0.2
	Airflow velocity (L/min)	4.0	3.5	3.5	4.0	5.0	4.0	6.0	3.0	4.1 ± 0.3
Arterial blood gas^b^	P/F	58	66	156	65	58	133	100	97	92 ± 13
	PH	7.4	7.4	7.4	7.4	7.2	7.3	7.3	7.4	7.3 ± 0.03
	PCO_2_ (mmHg)	55	42	45	34	58	45	91	49	52 ± 6
Disease severity score^c^	Murray lung injury	3	4	4	3	3	3	4	3	3.3 ± 0.2
	APACH E-II	19	11	20	21	19	23	23	18	19 ± 1
	SOFA	13	5	19	10	18	11	12	18	13 ± 2
Prognosis indicator	ECMO support days	39	46	21	16	9	36	39	46	32 ± 5
	ICU length of stay (days)	69	75	71	58	23	49	69	56	57 ± 8
	Outcome	Alive	Alive	Alive	Alive	Dead	Dead	Dead	Dead	/

*MV, mechanical ventilation; ECMO, Extracorporeal Membrane Oxygenation; P/F, PO_2_/FIO_2_; PCO_2_, Partial Pressure of Carbon Dioxide; APACHE, Acute Physiology and Chronic Health Evaluation; SOFA, Sequential Organ Failure Assessment; ICU, Intensive Care Unit*.

a *The day before ECMO;*

b *The day of starting ECMO;*

c *The day of admission to ICU*.

### Use of Sedative and Analgesic Drugs and Muscle Relaxants During ECMO Treatment

The sedative drugs used in this study were mainly midazolam and propofol. The analgesic drug was remifentanil, and the muscle relaxant was cisatracurium. The usage period was divided into three main time points, namely, the ECMO start date (Start), the ECMO support interim period (INTERIM) and the ECMO support end date (END). The specific dosages of the drugs are shown in Figure 1. Antipsychotic drugs were added to reduce the degree of sedation and analgesia in the four patients who were successfully taken off ECMO. Clonazepam 2 mg was administered orally three times a day, or olanzapine 5 mg was administered orally once a day.

The overall trend is that from the start of ECMO to the intermediate stage of ECMO support (Interim) (along with the prolongation of ECMO treatment), the doses of remifentanil, propofol and midazolam in eight patients were increased to different degrees, and the doses were generally high, resulting in a long treatment course. From the Interim ECMO support (INTERIM) to the end of ECMO, the doses of sedative and analgesic drugs used in the death group continued to increase, while the doses of the drugs were decreased in the survival group due to the disease remission in these patients and the improvement of the diseased lungs.

### Monitoring of the Depth of Sedation, Analgesia, and Muscle Relaxation

The degree of sedation was monitored using RASS and BIS (BIS value 0 stands for flat line; 0–40 indicates deep sleep and outbreak inhibition; 40–60 represents general anesthesia; 70 represents deep sedation; >70 represents mild to moderate sedation). The RASS scores for the two time points of ECMO Start and Interim ECMO support (INTERIM) were−5 and−5, respectively, and the BIS monitoring results were 58.5 (46, 76) and 79 (59, 89), respectively, both of which were deep sedations. The survival group gradually transitioned to a mild sedation plane before ECMO removal. The RASS score of the survival group was 1 at the end of ECMO, while the death group was still in a deep sedation state. The BIS monitoring results were 58.5 (46, 76), respectively. The RASS score was −5. The CPOT score was used for analgesic monitoring so that the CPOT score was <3 points. The muscle tone of the patients was monitored using TOF. The TOF monitoring values at the start of ECMO and the intermediate stage of ECMO support (Interim) were 65.6 (56, 70) and 77.5 (65, 86), respectively. The muscle relaxants were discontinued in the survival group and were decreased but not completely discontinued in the death group at the end of ECMO.

### Adverse Effects

The four patients who were successfully weaned from ECMO did not develop any severe adverse reactions. Three patients had different degrees of muscle tremors, with obvious facial manifestations, and one patient had pharyngeal paralysis and dysphagia. The patients who developed muscle tremors received subsequent TCM and rehabilitation physiotherapy, and their symptoms quickly improved.

## Discussion

The explanation of our finding: the treatment strategies may be slightly different due to the group responsibility system in our ward, but this does not affect the overall trend. In Group A, patient 2 did not receive propofol during the whole course, and our ECMO therapy was in an exploratory phase because this patient was the first to see the doctor, considering that propofol might affect the life of ECMO oxygenator. In Group B, patient 5 had a relatively mild illness, and midazolam was not used for ECMO at the beginning. However, due to the large dose of muscle relaxant, our attempt to reduce the dose of the muscle relaxant had failed, and our patient's target oxygen saturation could not be maintained. Hence, we were forced to use the muscle relaxant again, and midazolam was added to assist with sedation.

The muscle relaxants were used for much longer than 48 h. From the start of ECMO to the interim stage of ECMO support (INTERIM), the doses of muscle relaxants showed an increasing trend in both the death group and the survival group, except for two patients. Patient 1 did not receive muscle relaxants in the early stage, but in the late stage, it was difficult to reach the standard due to the worsening oxygen saturation. After a muscle relaxant was added, the patient's condition improved, and the dose showed an increasing trend. Patient 2, who was given small dose of muscle relaxant in the early stage and who did not receive any muscle relaxants in the middle and late stages, met the recommendations of the guidelines, but his oxygen saturation and blood pressure were always low, which made it was difficult to keep him alive. The four surviving patients in Group B progressed from the intermediate stage of ECMO support (Interim) to the end of ECMO with gradually reduced doses of the muscle relaxants until the muscle relaxants were eventually discontinued due to improvement in the patients' conditions. From interim ECMO support (INTERIM) to the end of ECMO (END), the doses of sedatives used in patient 1 and patient 4 in Group A increased with the prolongation of ECMO treatment. Patient's 3 muscle relaxant dose was gradually reduced because his lung condition was actually improving, and the muscle relaxant dose could be reduced. However, the patient eventually died due to complicated gastrointestinal bleeding.

The ELSO guidelines recommend minimizing sedation, analgesia, and muscle relaxation in patients with ARDS receiving ECMO support (8, 9). This study found that the sedative, analgesic and muscle relaxant strategies of patients with SARS-CoV-2 supported by ECMO were different from those in the ELSO guidelines, and the overall dose of these drugs was higher, resulting in a longer course of treatment. The doses of sedative, analgesic and muscle relaxants need to be continually increased while maintaining the same degree of sedation, analgesia and muscle relaxation. Four of the eight ECMO patients were successfully weaned from the ventilator, and the other four died due to an ineffective management of their complications. All four successful patients received relatively large doses of sedative and analgesic drugs as well as muscle relaxants. From the start of ECMO to the middle stage of ECMO support, the doses of sedative and analgesic drugs were increased instead of decreased in order to maintain a stable oxygen supply and consumption. This study suggested that deep sedation, analgesia and muscle relaxation could temporarily improve oxygenation, allow for treatment opportunities, give time to allow for the control of later primary disease, and could allow for the opportunity to wean the COVID-19 patients from ECMO when the patients developed uncorrectable hypoxemia or man-machine confrontation.

In this study, the doses of the sedative and analgesic drugs that we used were higher, and the course of treatment was longer. The cause of these findings were analyzed. The long course of treatment was related to the long course of SARS-CoV-2 itself. Only when the lung lesions improved could the use of sedative and analgesic drugs be reduced. Attempts to reduce the depth of sedation and analgesia during the course of the disease were unsuccessful because of our patients' restlessness and the difficulty in maintaining the oxygen saturation of the patients at an ideal level. In addition, the doses of sedative and analgesic drugs are not reduced with the usual process, but the dose demand is gradually increased. The main reasons were considered to be the following: doctors and nurses wearing protective clothing and goggles played a certain role in blocking the observation of the patient's condition and the catheters were easily pulled out due to the shallow depth of sedation. Our data show that the depth of sedation in the middle stage of ECMO treatment was still deep sedation, which was inconsistent with the guidelines, and this was related to the inability to reduce the depth of sedation and analgesia during the actual operation of ECMO. From the perspective of the doses of sedative and analgesic drugs used, the doses of these drugs were increased without changing the degree of the sedative and analgesic effects, and the BIS score was not decreased. An *in vitro* study by Shekar et al. demonstrated for the first time that there was a significant reduction in midazolam and fentanyl in the circuit of 24-h extracorporeal membrane oxygenation in adults (10). This may partly explain the increased dose requirements for these sedative and analgesic drugs during ECMO.

For ARDS patients supported by ECMO, the use of muscle relaxants can reduce ventilator-associated lung injury, and 25–45% of ARDS patients receive neuromuscular blockers (NMBAs), with an average time of 1 2 days (11). In this study, all eight patients were treated with muscle relaxants at doses greater than the regular dose and for a longer period of time. We also tried to reduce the dose of the muscle relaxants or even stop using them, but once the dose was reduced, the patient immediately developed tachypnea, ventilator resistance and difficulty in maintaining the peripheral oxygen saturation. During this time, the doses of the sedative and analgesic drugs were already relatively high, and the P/F ratio could be improved after the dose of muscle relaxants was again increased. In our analysis, we considered that patients with SARS-CoV-2 had severe lung lesions and long ECMO support times, so the course of treatment for muscle relaxants was long. In addition, severe patients often suffer from obvious abdominal flatulence, and their diaphragm moves upwards. On the one hand, this will affect lung ventilation and aggravate hypoxia. On the other hand, abdominal distension can easily affect the flow rate of ECMO, and an unstable flow rate will lead to decreased oxygenation. Studies have reported that when hypoxia occurs, adequate sedation and analgesia and muscle relaxation can reduce spontaneous respiratory movement, thus reducing the oxygen consumption (4). In addition, in this study, it was also found that the degree of muscle relaxation in the intermediate stage of ECMO support (Interim) was shallower than the start date of ECMO. However, the dose of muscle relaxants was actually increased instead of decreased. These results suggest that the pharmacodynamics and pharmacokinetics of muscle relaxants may also be altered by ECMO.

ECMO may cause significant changes in the pharmacokinetics (PK) of a drug in three ways (12, 13): (I) the retention of the drug in the ECMO lines; (II) an increase in the apparent volume of distribution (Vd); and (III) a decreased drug clearance. The drug retention is affected by the oxygenator material, the type of conduit, the life of the circuit, and the composition of the prefilled fluid. The ECMO circuit includes a conduit and a membrane oxygenator that adds additional body surface volume, and the drug is trapped in the circuit, resulting in an increase in the apparent volume of distribution and a decrease in the plasma drug concentration (12, 14). ECMO may change the apparent volume of distribution (VD) of drugs through the following mechanisms: (I) drug retention; (II) hemodilution; and (III) ECMO-related physiological changes. It is common for critically ill patients treated with ECMO to develop PK changes associated with systemic inflammatory response syndrome, which may lead to an increase in the Vd of hydrophilic drugs (15). In addition, significant changes in the blood pH may occur in patients receiving ECMO therapy, resulting in further changes in the drug distribution, ionization level, and protein binding (16). It should be noted, however, that much of the PK data in the ECMO setting are from neonates (17), and extrapolation from these data must be used with caution due to the differences in the neonatal immature glomerular and renal tubular function as well as the developing liver function (18). In general, the plasma clearance (CL) of drugs was lower in ECMO patients than in those not treated with ECMO (14) due to the hepatic and renal hypoperfusion and hypoxemia (19). The effects of the decreased drug clearance were partially offset by an increased cardiac output due to an initial inflammatory response, aggressive fluid therapy, and positive inotropic drug use (15).

## Conclusion

When patients with SARS-CoV-2 receive adjuvant support with VV-ECMO, they require special protection and isolation, and their disease may involve multiple systems, resulting in refractory hypoxemia. In addition, the pharmacokinetics of drugs are changed, which requires the use of increased doses of sedative and analgesic drugs, and the time while using these drugs is relatively long. Therefore, the PK of the drugs should be considered in the diagnosis and treatment of these patients, and individualized titration and adjustment should be performed in combination with the actual clinical situation.

## Data Availability Statement

The original contributions presented in the study are included in the article/supplementary material, further inquiries can be directed to the corresponding author/s.

## Author Contributions

YL and YG conceived this research. FW, ML, ZZ, and JS collected data. FW and ML completed the statistics. FW and YG made the table and figure. FW and YG completed the writing of the full text of the paper. YL reviewed the entire paper. All authors approved it for publication.

## Conflict of Interest

The authors declare that the research was conducted in the absence of any commercial or financial relationships that could be construed as a potential conflict of interest.

## Publisher's Note

All claims expressed in this article are solely those of the authors and do not necessarily represent those of their affiliated organizations, or those of the publisher, the editors and the reviewers. Any product that may be evaluated in this article, or claim that may be made by its manufacturer, is not guaranteed or endorsed by the publisher.
